# Global and local environmental changes as drivers of Buruli ulcer emergence

**DOI:** 10.1038/emi.2017.7

**Published:** 2017-04-26

**Authors:** Marine Combe, Camilla Jensen Velvin, Aaron Morris, Andres Garchitorena, Kevin Carolan, Daniel Sanhueza, Benjamin Roche, Pierre Couppié, Jean-François Guégan, Rodolphe Elie Gozlan

**Affiliations:** 1 Centre IRD de Montpellier, Département Santé, UMR MIVEGEC IRD-CNRS-Université de Montpellier, 34394 Montpellier, France; 2 The Royal Veterinary College, Department of Production and Population Health, The Royal Veterinary College, Hawkshead Lane North Mymms, Hatfield, Hertfordshire AL9 7TA, UK; 3 Department of Global Health and Social Medicine, Harvard Medical School, Boston, MA 02115, USA; 4 PIVOT, Division of Global Health Equity, Brigham and Women's Hospital, Boston, MA 02115, USA; 5 Computational & Systems Biology, Rothamsted Research, Harpenden AL5 2JQ, UK; 6 UMMISCO, Département Sociétés et Mondialisation, UMI IRD-UPMC 209, 93143 Bondy, France; 7 Université de Guyane, EA3593 Epidémiologie des Parasitoses Tropicales, 97306 Cayenne, French Guiana, France; 8 Service de Dermatologie, Cayenne Hospital, rue des Flamboyant, BP 6006, 97306 Cayenne, French Guiana, France; 9 Future Earth International Programme, OneHealth Global Research Project, Future Earth Montréal Hub, Montréal, QC H3H 2L3, Canada; 10 Institut de Recherche pour le Développement, Département Ecologie, Biodiversité et Fonctionnement des Ecosystemes Continentaux, UMR BOREA IRD 207, CNRS 7208, MNHN, UPMC, Muséum National d'Histoire Naturelle, 75231 Paris, France

**Keywords:** aquatic ecosystem, climate, generalist pathogen, *Mycobacterium ulcerans*, tropical

## Abstract

Many emerging infectious diseases are caused by generalist pathogens that infect and transmit via multiple host species with multiple dissemination routes, thus confounding the understanding of pathogen transmission pathways from wildlife reservoirs to humans. The emergence of these pathogens in human populations has frequently been associated with global changes, such as socio-economic, climate or biodiversity modifications, by allowing generalist pathogens to invade and persist in new ecological niches, infect new host species, and thus change the nature of transmission pathways. Using the case of Buruli ulcer disease, we review how land-use changes, climatic patterns and biodiversity alterations contribute to disease emergence in many parts of the world. Here we clearly show that *Mycobacterium ulcerans* is an environmental pathogen characterized by multi-host transmission dynamics and that its infectious pathways to humans rely on the local effects of global environmental changes. We show that the interplay between habitat changes (for example, deforestation and agricultural land-use changes) and climatic patterns (for example, rainfall events), applied in a local context, can lead to abiotic environmental changes and functional changes in local biodiversity that favor the pathogen's prevalence in the environment and may explain disease emergence.

## INTRODUCTION

Emerging and re-emerging infectious diseases (EIDs) are rapidly increasing in incidence, thus resulting in locally severe consequences for humans, wildlife and overall ecosystem health.^1^ Many of these diseases are caused by generalist multi-host pathogens that infect and transmit via multiple host species.^2^ Owing to the existence of multiple dissemination routes within the environment, understanding of their transmission pathways to humans is often limited.^2^

In addition, global environmental changes, defined here as land-use changes, climatic patterns or biodiversity alterations taking place at a local level, contribute to EIDs through effects on abiotic environmental conditions, host–pathogen interactions (for example, changes in the local abundance of suitable hosts/pathogens) or transmission pathways. In tropical developing countries, such environmental modifications are locally revealed through, for example, a change in rainfall patterns, an increased level of deforestation or expansions in agricultural land, new roads and dams modifying the structure of floodplains and an overall increased level of urbanization underpinned by rapid socio-economic changes and human population expansion. These effects are exemplified at a local level by rapid shifts in ecosystem function, thus creating opportunities for generalist pathogens to exploit new ecological niches and infect new host species, thereby modifying their transmission pathways.^2, 3^

**Figure 1:**
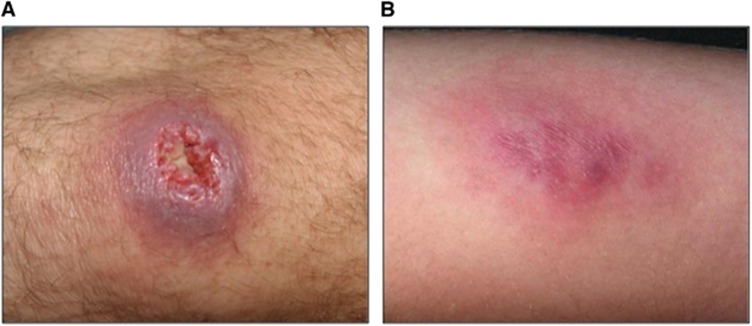
Two types of lesions caused by Buruli ulcer (BU). (**A**) An ulceration of the knee; (**B**) A plaque on the arm. These photographs were provided by Professor Pierre Couppié (Cayenne Hospital), who has an ethical agreement with the patients.

**Figure 2:**
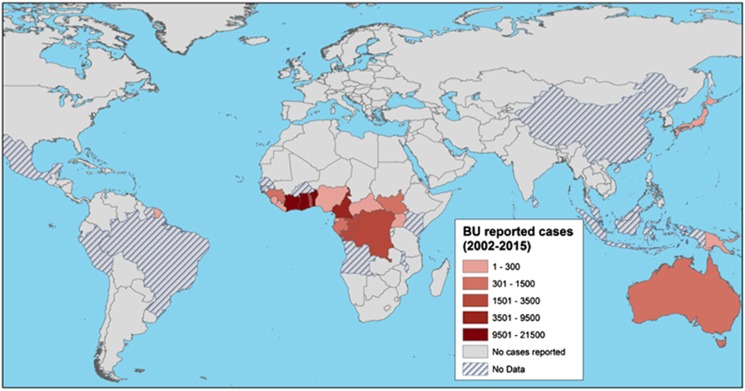
Global distribution of Buruli ulcer (BU) cases, from 2002 to 2015. The map displays the total number of BU cases reported to the World Health Organization in 2016 on a red color scale.^8^ This information was completed for French Guiana by collaborators, on the basis of hospital records (Professor Pierre Couppié, personal communication). The map was created using ArcMap v.10.2.2.

An example of disease emergence suspected to have occurred because of such global environmental changes is Buruli ulcer (BU). BU is a human necrotizing skin disease that was first detected in 1937 in Southeast Australia^4^ and in 1942 in Eastern tropical Africa.^5^ The clinical signs of the disease include painless nodules, plaques and edema, and subsequent development of skin ulcers that may have devastating effects on the human host and eventually result in osteomyelitis and permanent disability if early detection and proper treatment are delayed or absent (Figure 1).^6, 7^ BU annually affects between 5000 and 6000 cases across 33 countries worldwide (Figure 2), mainly in humid tropical and subtropical areas, with significantly higher numbers of cases on floodplains, where humans have contact with slow-moving or even stagnant contaminated water bodies (for example, rivers, ponds, swamps and lakes).^7, 9, 10^ African countries are the most affected, and the highest incidence rates are found in Benin, Côte d'Ivoire and Ghana; in some areas, such as Ghana or Benin, BU is even more prevalent than tuberculosis and leprosy.^11^ Since the 1980s, the incidence of cases has significantly increased in West African countries, where children 5–15 years of age are the most affected in endemic regions in terms of the incidence and severity of the disease.^12^ BU mainly affects poor rural human populations living in tropical regions and imposes significant economic and public health burdens (for example, impoverishment, cost of illness and loss of productivity)^13^ in endemic areas and has therefore been categorized by the World Health Organization (WHO) as one of the 17 neglected tropical diseases.^11^

Two of the key research priorities determined by the Global BU Initiative (GBUI) initiated by the WHO in 1998 have been to clarify and identify (i) the ecology of BU and its causative agent *Mycobacterium ulcerans* (MU) and (ii) the mode(s) of transmission of the bacterium from the environment to humans. Despite nearly 20 years of research, the exact mode(s) of transmission to humans and the etiology of BU remain poorly understood, primarily because many cases are characterized as ‘sporadic and unpredictable'.^6, 14^ However, multiple studies have focused on characterizing the MU ecological niche in different natural settings where the disease is endemic, and have provided substantial new evidence regarding the ecology, epidemiology and mode(s) of transmission.

Here we review the global to local ecological factors that underpin the emergence of BU and describe the anthropogenic (for example, land-use changes) and climatic framework that affects disease transmission pathways. Specifically, we show that infections are probably driven by a combination of human behaviors, climatic drivers and changes in ecosystem function that affect abiotic and biotic environmental conditions in aquatic ecosystems and local biodiversity. The literature clearly shows that the transmission of MU from the environment to humans proceeds through multiple environmental pathways responding to the same global ecological rules but with local drivers.

### Transmission dynamics of MU in the environment

#### Evidence for the multi-host transmission of MU

Many field studies conducted in countries where BU cases have been reported have detected MU DNA from diverse aquatic ecosystems and from a high diversity of taxa, representing a wide range of potential environmental reservoirs and/or intermediate hosts for the mycobacteria (Table 1).^6^ These observations thus suggest that multiple hosts can become contaminated from the aquatic environment, and some of them potentially play a role in the persistence of MU in this habitat. Although pioneering studies have mainly been performed from a microbiological perspective, in the past several years, several authors have focused on examining the role of the whole aquatic species community. Recent ecological studies^15, 16, 17^ and mathematical models^18, 19^ have markedly improved knowledge of the ecological niche of MU and have clearly shown its multi-host transmission dynamics. As presented in Table 1, a vast array of taxa can acquire and then transmit MU through ecological networks. Aquatic communities, for example, are composed of organisms with very different functional feeding strategies (for example, filtering collectors, gathering collectors, scrapers, scavengers and predators). This diversity of functional traits might yield multiple transmission pathways to infection,^19^ thereby suggesting that the mycobacteria might passively disseminate through the entire aquatic community via trophic or organism-to-organism contact links.^18^ Moreover, some infected terrestrial mammals (for example, possums and mice) have been found to harbor MU,^20, 21^ possibly from living close to an infected water body and becoming infected either during watering or feeding, or even by an insect bite.

**Figure 3:**
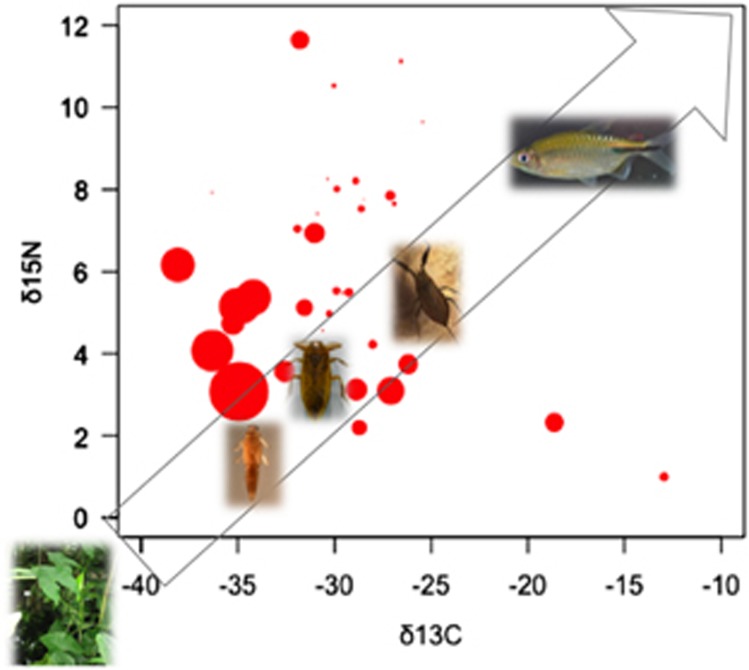
Distribution of *Mycobacterium ulcerans* (MU) DNA across the entire aquatic food web. Organisms assimilate carbon (C) and nitrogen (N) stable isotopes by feeding, and analysis of their stable isotope signatures thus allows for the study of trophic food webs. The direction of the arrow and the pictures of some MU hosts illustrate the food web, from low to high trophic levels: aquatic plants (Araceae), Baetidae, Belostomatidae, Nepidae and fish (Cichlidae). The average δ^13^C and δ^15^N signatures were obtained from stable isotope analyses of each taxon of the aquatic environment sampled from 17 sites in French Guiana (South America) and tested positive by qPCR for IS*2404* and KR genetic markers.^22^ The amplification of these markers confirms the presence and abundance of MU. For each taxon, the average bacterial load (for example, number of bacteria per mg of organism) is represented by the size of the red circles on the basis of transformed data using the square root mean number of bacteria (detailed information in Supplementary Table S2). Adapted from Morris *et al.*^22^

In line with these observations, a recent field study conducted over 17 distinct sites in French Guiana (South America) and from a range of taxa representing the entire aquatic community has shown the dissemination of MU via trophic links.^22^ On the basis of stable isotope analysis (that is, an estimation of the trophic level of a taxon) and estimations of the mean bacterial load (that is, bacteria per mg of organism, from DNA quantification by qPCR) for each taxon, it has been confirmed that this generalist pathogen passively transmits via the aquatic food web by biological interactions (Figure 3). Although MU DNA can be found in the whole aquatic community, these results suggest that low trophic level organisms (for example, filter feeders and scavengers) might acquire the bacterium from sediments, mud, detritus, plant and algae biofilms where MU preferentially flourish^21, 23, 24, 25^ and even from chitinous remains.^26^ However, because low trophic level organisms show greater bacterial loads, the passive transmission of MU by contact with intermediate (for example, taxa Baetidae, Caenidae, Leptophlebiidae and Corixidae) and higher (for example, Tanypodinae and Coenagrionidae) trophic level organisms, increasing contact rates with humans through daily activities (for example, swimming, bathing and fishing), appears to be more likely than (for instance) its bio-accumulation by specific aquatic insects, as has previously been proposed.^6, 27^

### How biological interactions influence the environmental dynamics of MU

The multi-host transmission dynamics of MU via trophic links suggests that the biodiversity and food web structure in aquatic sites might play a major role in the transfer and abundance of the bacteria in the environment. At the community level, Benbow *et al.*^16^ have shown an association between aquatic macro invertebrate community structure and MU-positive sites in Ghana (Africa). They have found four aquatic macro invertebrate taxa, such as Hemiptera (Pleidae, Gerridae), Odonata (Libellulidae) and Hydracarina (water mites), to be significant indicators of the bacteria's presence in the environment. In addition, Morris *et al.*^15^ have recently shown that shifts in host functional diversity (for example, changes in host species that have similar functional traits) further characterize the ecological niche of MU, and there is a specific association with gathering collectors and filter feeders. Finally, metacommunity analyses have suggested that, in lotic systems (for example, flowing waters), stochastic biodiversity loss (for example, random extinction of taxa due to significant water flow) restructures the ecological networks by which MU disseminates, thus leading to a lower MU prevalence.^17^ In contrast, in lentic systems (for example, stagnant waters), niche-based processes, such as the turnover of aquatic taxa, govern biodiversity changes (for example, extinction of taxa and persistence of functional hosts) lead to a greater MU prevalence.^17^ Consequently, it has been suggested that some host species may act as amplifiers of MU in the environment and boost its prevalence, whereas others act as diluters and decrease its prevalence. These results show that collapses in the aquatic food web structure, whether anthropogenic (for example, deforestation) or natural (for example, changes in weather), notably in floodplains, may lead to a shift in functional diversity, thereby resulting in major changes in ecosystem function and providing new lentic environments favoring the bacterial growth.^15^ For instance, vacant niche spaces after deforestation in flooded tropical forests would rapidly change the light levels, water temperature, pH and oxygen levels. These abiotic changes would in turn enhance the persistence of MU as free-living in the environment and cause rapid replacement of local functional biodiversity, a condition that also favors MU and suitable host development. These local shifts in functional diversity would increase the infectious risk when they occur in direct proximity to human populations.

An ecological perspective based on specific functional traits rather than taxonomic groups of organisms may strengthen understanding of the environmental drivers of generalist pathogens and their efficient transmission to humans.^15^ Whereas a loss of biodiversity due to environmental disturbances may not necessarily trigger the emergence of generalist infectious pathogens, a local shift in functional host diversity creates new ecological niches available for a variety of functionally close species able to harbor such pathogens.^15, 28^

### Transmission of MU from the environment to humans

Despite the improved understanding of global and local drivers of MU in the environment, the transmission to humans remains unclear, probably because of the presence of the many potential transmission routes typical of generalist pathogens. Different potential transmission mechanisms of MU from the environment to humans have been proposed: (i) airborne transmission through contamination with atmospheric aerosols from the surfaces of stagnant waters, (ii) environmental transmission involving the non-specific inoculation of MU by direct contact with skin lesions or injuries and (iii) vector-borne transmission by biting aquatic insects.^6, 29^ After the first identification of MU in Hemipteran aquatic insects (for example, water bugs) by Portaels *et al.*,^30^ many experimental studies have focused on the roles of these insects as potential vectors in the transmission of MU from the wild to humans, particularly those from the Naucoridae and Belostomatidae families.^23, 27, 31, 32, 33^ Whereas numerous studies have indicated that MU may be transmitted to humans via aquatic insect bites, the possibility of purely vector-borne transmission is still criticized. Some authors have argued that water bugs bite humans only sporadically, and have questioned whether such transmission could account for all BU cases in endemic areas; moreover, epidemiological studies in the past several decades have not found insect bites to be a consistent risk factor for BU.^6^ Field studies have shown that MU is ubiquitous in both endemic and non-endemic regions, that the entire aquatic community of animals and plants serve as potential intermediate hosts and/or environmental reservoirs,^6, 24^ and that multiple transmission pathways of MU exist through food webs and/or biological interactions.^19, 32^ In this context, evidence for a specific role of water bugs in BU transmission from ecological field studies is still ambiguous. Thus, an interesting question is whether aquatic hemipterans represent the only transmission pathway of MU or whether various routes of transmission exist, with contributions to the burden of BU depending on the local context.

In line with this scenario, Garchitorena *et al.*^29^ have shown that in BU endemic regions of Cameroon, proxies of MU environmental load consistent with a non-specific environmental transmission pathway better explain the spatial and temporal patterns of BU incidence in human populations rather than exclusive vector-borne transmission through water bugs. Interestingly, direct inoculation into the skin seems necessary for MU to be transmitted,^34^ thus suggesting that passive contamination through pre-existing wounds is not a viable pathway. However, inoculated bacterial loads necessary to trigger BU infection are very low (10–10^3^ in mice),^31^ and thus any MU-contaminated environmental reservoir able to puncture or lacerate the human skin could potentially inoculate MU into the dermis and lead to BU infection. Indeed, in some cases, MU can be inoculated by the bites of various insect species (not only Hemipterans) and, in other cases, can be mechanically inoculated through contact with aquatic plants (for example, cuts) or even insects harboring the bacilli externally.^35^ For instance, Lavender *et al.*^36^ have found a positive correlation between the proportion of MU-positive mosquitoes and BU incidence in Australia, and the bacteria's DNA has been detected on the external parts of the body (for example, the exoskeleton and legs) of adult mosquitoes.^37^ Furthermore, although Zogo *et al.*^35^ have found that, in Benin (Africa), terrestrial flying insects do not harbor MU DNA, contradictory results have been found in Cameroon, where terrestrial insects collected from rural and urban houses in a BU endemic region have been found to be positive for MU DNA.^38^ These results, in addition to the multi-host feature of MU within the environment, clearly suggest multiple transmission pathways from the environment to humans that respond to the same ecological rules but are influenced by local drivers. In other words, two people at the same location can be infected by MU in different ways although the distribution of the pathogen in the environment responds to the same ecological dynamics. This phenomenon suggests new research directions to be explored, and future work should investigate the global drivers of local conditions that promote these various transmission pathways.

Human infections can thus result from the interaction of multiple factors such as (i) socio-economic factors driving behaviors and practices that favor environmental conditions for MU and the exposure of humans to MU foci, (ii) differences in the pathogenicity of MU strains (for example, the strain diversity in Box 1) and (iii) genetic factors and local exposure influencing immune responses in the human host. First (i), the burden of BU is concentrated in poor rural areas of sub-Saharan Africa, where access to safe water and sanitation is low.^39^ Therefore, local populations in these regions often use stagnant and slow-flowing bodies of water for daily activities such as laundry, personal hygiene and recreation, which have been previously found to be risk factors for the disease.^40, 41^ Interestingly, Vincent *et al.*^42^ have found a contrasting over-representation of boys among younger patients and of women among older patients, a pattern that may be explained by the different age- and gender-related habits in aquatic sites, such as rice planting and harvesting in many countries of Western Africa, which are mainly done by women and children. Furthermore, socio-cultural factors may also explain local environmental changes that have caused an increased risk of BU transmission in endemic areas.^7^ For instance, macro-economic fluctuations in agricultural subsidies, population increases and social rupture are thought to be responsible for the extensive deforestation near the basin of the Nyong River (Cameroon), with subsequent BU expansion in the region.^7^ Second (ii), the small number of cases in South American countries, such as Peru^43^ or French Guiana,^44^ compared with Africa,^12^ appear to be associated with a lower virulence of non-African MU strains and possibly with lower water-use habits compared with those observed in Africa. Indeed, the two types of mycolactone (for example, the molecule responsible for extensive lesions in vertebrate hosts, including humans) isolated from African strains are more cytotoxic than the mycolactones produced by the dominant strains elsewhere.^45^ These findings were later confirmed by Ortiz *et al.*,^46^ who have observed variable local immune responses in mice depending on the infecting strain, with African strains inducing the most severe inflammation, necrosis and bacterial loads.^46^ Third (iii), variable genetic susceptibilities and/or protective immune responses in humans may further influence the observed patterns of BU persistence. For instance, in endemic regions of Benin (Africa), healthy patients show higher antibody titers against salivary proteins of blood-feeding aquatic insects, as compared with patients who develop BU, thus suggesting that individuals experiencing a persistent insect bite are less likely to develop the disease because of potential immunization.^23^ In addition, initial evidence suggests that HIV infection can increase the risk and severity of BU, probably as a result of the underlying immune suppression in HIV cases.^47^

### Effects of land-use changes on Buruli ulcer emergence

Rapid human demographic increases in developing and low-income countries has resulted in significant pressures on local water resources and on the freshwater ecosystem as a whole, thereby leading to changes in the epidemiological patterns of water-borne infectious diseases.^15^ However, the roles of anthropogenic ecological changes in the emergence of environmental pathogens remain poorly understood.

Soon after its discovery, MU was identified as an environmental mycobacterium. Many epidemiological studies conducted in different tropical countries have linked BU cases with the proximity of infected human populations to slow-moving or stagnant water sources, such as floodplains or swampy areas,^6, 14, 48, 49^ thus suggesting that water might be the primary source of infection.^50^ As early as 1975, the WHO reported that the incidence of BU in Benin was 10 times higher in areas that have undergone environmental disturbances than in controlled areas.^14^ However, Ross *et al.*^51^ first hypothesized that local environmental disturbances close to Victoria (Australia) might have provided a suitable environment for MU to flourish. Specifically, they found that 28 out of the 29 BU patients studied were staying near or frequently visited the stagnant water bodies that appeared after the construction of a road across a swamp between 1991 and 1992.^51^ Other observations have linked BU/MU occurrence to environmental disturbances resulting from (i) deforestation practices and land-use changes (for example, agriculture), (ii) flooding of lakes and rivers during heavy rainfall, (iii) eutrophication, (iv) dam construction to create impoundments and wetlands, (v) construction of agricultural irrigation systems and rice fields and (vi) population settlement and encroachment close to water bodies.^6, 52^

Although the ecological association between BU/MU systems and human-disturbed aquatic environments has more often been anecdotal and related to specific human activities,^6^ recent studies in the central and southwestern regions of Côte d'Ivoire (Africa) have modeled these associations. On the basis of spatial mapping analyses, Brou *et al.*^52^ have found that the highest disease prevalence occurs where the presence of dams favor agricultural activities (for example, rice-fields, banana crops) in bordering deforested areas with a year-round wet climate. These findings have further been confirmed in Benin and Ghana (Western Africa), where the BU prevalence has increased with rural agricultural land use close to contaminated and stagnant water sources and has decreased with urbanization.^53^ In Benin, Côte d'Ivoire and Cameroon, decreased distance between agricultural fields and rivers has been found to be a risk factor for BU disease emergence.^48, 52, 53^ The effects of deforestation and land-use changes can be found in habitats surrounding villages with a high BU incidence.^54^ Moreover, in Australia, Hayman^50^ has found a relationship among BU disease, deforestation and erosion (for example, extensive bushfires) that might have disrupted the borders of the rainforest, as also reported in Africa.^55^

In addition, local needs to control flooding and provide electricity have led to the construction of dams in many tropical countries. The consequences of dam construction are rapid and have large effects on the ecological functioning of floodplains. In regulating river discharges, dams also tend to reduce downstream flooding, thereby reducing the seasonal structure of oxbows. Although they are considered drivers of environmental changes, the local effects of dams can be contradictory and in some places may leave a settlement of disconnected lentic oxbows in downstream floodplains, thus favoring the emergence of BU cases.^25^ In other places, such as in French Guiana (South America), dams decrease the seasonal flooding of downstream districts with the drying out of the floodplain, thus reducing the amount of favorable MU habitats.^44^ In French Guiana, for example, after the Petit-Saut Dam construction, a significant decrease in BU cases was observed downstream, with 10.1 versus 0.6 cases per 100 000 people per year, respectively.

In tropical areas, all these habitat modifications might disrupt the borders of the rainforest, change the floodplain topography and create swampy areas. During flooding and heavy rainfall events, the mycobacterium present in aquatic environments can be washed into and contaminate run-off water. During the dry season, water recedes from flooded habitats, thus leading to the formation of small water bodies (for example, oxbows and disconnected channels). Such rapid transformations of the local ecosystem lead to a converging new ecological niche characterized by water stagnation, increased light levels in surface water and higher water temperatures. These changes further lead to sedimentation (for example, turbidity), decrease the ultraviolet light, oxygen and pH in the water column and favor macrophyte growth and algal biofilm formation.^6^ As described above, such major changes in abiotic factors appear to provide environmental conditions that allow for the persistence of free-living stages of MU or enhance its growth.^6, 19^ Moreover, these physico-chemical changes might affect the aquatic community composition and suitable MU hosts, thus resulting in a turnover of host species communities from communities functionally adapted to lotic habitats to new communities with traits better adapted to lentic habitats. Therefore, these local ecosystems prone to MU, along with agriculture intensification, human settlement and field works, may increase the risk of contact with and transmission to humans.^7, 54^

The literature reviewed here leaves no doubt about the roles of anthropogenic land-use changes as drivers in the emergence of BU at the local level. However, the geographical variability in the number of BU cases and the severity of the disease cannot be explained solely by population settlement and encroachment. Although increased urbanization might provide higher levels of socio-economic and health infrastructure—allowing, for instance, the use of treated water and access to care—in rural areas, population expansion associated with poverty and unsanitary conditions may increase the risk of contact with environmental infectious pathogens.^48, 53^

### The role of spatio-temporal climatic patterns in Buruli ulcer emergence

The link between BU and tropical climatic patterns was first reported by Barker,^56^ who connected an increase in the number of BU cases in Uganda (Eastern Africa) between 1962 and 1964 with heavy rainfalls that flooded Lake Victoria. Soon thereafter, Radford^57^ reached a similar conclusion about the rise of BU cases in human population settlements on the riverbank and marked flooding events. In both reports, although the disease was associated with flooding events, BU cases occurred during the subsequent dry season, and this lag phase was attributed to the bacteria's incubation period (that is, the time between exposure to the pathogen and the appearance of clinical signs), now estimated to be between 3 months, on the basis of data reported in Uganda, and 4.5 months, on the basis of data reported in Australia, thus resulting in a delay between infection and diagnosis.^10^

Since then, long-term time series of BU cases and climatic models in Australia, South America and Central Africa, have revealed a robust correlation between disease incidence and seasonal climatic changes.^10, 58, 59^ In Cameroon, Landier *et al.*^10^ have found that seasonal flooding of the Nyong River created temporary swamps associated with a higher prevalence of BU. In particular, a significant association has been found between BU cases and rainfall patterns in the short term (for example, 6 months) and the long term (for example, decades), with the disease being reported after periods of heavy rainfall and flooding of the floodplain.^58, 59^ BU cases occurred more frequently during the dry season that followed a wet period. In French Guiana (South America), Morris *et al.*^59^ have linked stochastic BU cases with climatic anomalies, such as La Niña events, that are responsible for unusually dry periods during the rainy season.

The close link between rainfall patterns and BU cases suggests that seasonal changes in ecological functioning of the aquatic ecosystems and MU ecology affect the risk of transmission to humans. Indeed, MU environmental fluctuations during the year show similar seasonal patterns in Cameroon,^9, 33^ and cumulative precipitation in these areas is associated with a higher MU prevalence in aquatic ecosystems, even after controlling for seasonal changes in biotic and abiotic factors.^19^ Moreover, although the presence of MU has been more frequently detected in still lentic (for example, swamp) than in flowing lotic (for example, river) systems,^9^ seasonal variations in MU presence vary widely depending on the type of ecosystem. In Cameroon, for instance, MU exhibits major seasonal and intra-seasonal variations in temporary flooded areas and large rivers, whereas the bacteria's presence is less variable between seasons in permanent swamps and streams.^9^ Furthermore, Carolan *et al.*^49^ have suggested that, during the dry season, MU is more likely to be present in small streams near urban and agricultural areas. Although MU appears to be ubiquitous, a pattern emerges from the literature review, in which heavy rainfalls lead to erosion of floodplain soils, and flooded habitats area precursor of MU redistribution across wetland ecosystems. As described above during the dry season, or drier decades, as shown by Morris *et al.*,^59^ water recedes from flooded habitats, which (in association with deforestation and land-use changes) creates stagnant water bodies prone to rapid local abiotic changes (for example, higher temperature, biofilm proliferation, lower pH and lower oxygen) along with a turnover of the biotic community.^17, 49, 59^ These lentic habitats provide ecological conditions that may favor MU persistence, growth and transmission, and many suitable aquatic hosts.^19^

Whereas the emergence of BU disease has been long characterized as unpredictable, recent studies conducted in different countries show that the interaction between certain climatic patterns and land-use changes (for example, deforestation and agriculture practices) can predict the number of BU cases and may thus predict the risk of infection.^10, 59^ As discussed here, the interplay between deforestation (changes in landscape ecology and topography) and rainfall events (flooding) allow the redistribution of MU in new environments where abiotic and biotic conditions enhance its prevalence and increase contact rates with human hosts. In addition, at the start of the dry season, these lentic habitats become more accessible for a whole range of human activities, such as crab hunting, fishing, bathing and washing clothes. However, further work must be performed on a smaller scale to better model the effects of rainfall and abiotic changes on the distribution of MU strains and other mycobacteria that may potentially compete with MU at the local level. Although the bacterial load tends to increase toward the start of the dry season, the mechanisms linking the bacteria's distribution to human cases, e.g., transition to disease, must be further investigated. Here, on the basis of the literature review, we propose that climatic events together with land-use modifications synchronize changes in the ecological function of the wetland ecosystem that favor the development of the bacteria and, along with changes in human activity, increase contact with nearby infected water sources. Future research should focus on the prediction of BU infection risk established on the basis of a set of local socio-economic and climatic scenarios.

### Box 1. Genetic diversity among MU strains

Phylogenetic analyses suggest that all MU strains emerged from a common *M. marinum* progenitor, which infects fish and can occasionally cause cutaneous disease in humans.^60^ Whereas previous genetic comparisons of MU strains using genome microarray analysis have indicated a distinction between two main lineages worldwide, called ‘classical' and ‘ancestral' lineages, and six continental haplotypes (for example, African, Australian, South-East Asian, Asian, South American and Mexican strains),^61^ MU has been long described as a monomorphic species with a restricted genetic diversity.^62^

Despite this continental differentiation, the lack of reliable genetic markers and culture methods to isolate MU strains from the environment have constrained the distinction between isolates within the same geographic area,^62, 63^ thus limiting the understanding of MU transmission pathways; high-resolution genetic typing of the different strains co-circulating in the environment are thus needed.^64^ Although pioneering work by Stragier *et al.*^65^ and Ablordey *et al.*^66^ has indicated low heterogeneity among MU isolates from Africa, further genome sequencing has identified Variable Number of Tandem Repeat (VNTR) regions in the MU genome (for example, genome regions exhibiting polymorphisms on the basis of different numbers of tandem repeat motifs). This discovery has allowed for differentiation between MU and other mycolactone-producing mycobacteria (MPM) and has revealed genetic variability among clinical African strains,^62, 63^ thus suggesting that matching VNTR profiles of environmental and clinical strains of MU from the same geographic area should allow for the identification of its transmission pathways.^24^ Similarly, Single-Nucleotide Polymorphism (SNP) typing assays have identified multiple strains co-circulating within the same regions in Ghana (Africa), which were descendants of founder genotypes that have spread over the regions, thus representing focal transmission clusters.^64, 67^

Williamson *et al.*^24^ have found that MU is widely distributed in both endemic and non-endemic regions of Africa, and thus the ubiquity of this pathogen in the environment contrasts with the local distribution of BU cases, which occur within specific geographic villages within endemic regions.^24^ Furthermore, sero-epidemiological studies have shown that, although an important proportion of the human population living in endemic regions is exposed to MU, only a small proportion develops the disease.^68^ These results suggest, for instance, that although PCR methods might detect all strains of MU in the environment, only some specific strains might have the potential to cause disease in humans. Alternatively, some sites may harbor the higher bacterial loads necessary to cause disease in humans. In French Guiana (South America), by using multiple VNTR markers, Reynaud *et al.*^69^ have found high genetic variability among clinical isolates of MU that has allowed for the identification of three genotypes. Furthermore, in Ghana (Africa), Narh *et al.*^21^ have tracked MU infections from contaminated environments by typing strains isolated from humans and from the environment. These authors have found four genotypes present in humans and in environmental samples, as well as three additional genotypes observed only in soil and biofilms. This study has provided the first evidence of the existence of several genotypes co-circulating in the environment and has suggested that only some of them might be pathogenic for humans (possibly because of specific genetic characteristics or even mycolactone type). Although this recent discovery has clearly improved understanding of the genetic diversity among environmental strains of MU, their spatio-temporal distributions as well as their ecological niches within the local communities of host species of MU are lacking and still impede the characterization of their transmission routes to humans.^21^ Indeed, as reviewed here, MU disseminates through multiple host species within the environment, and some of them maybe key host species favoring contact with (and transmission to humans of) these infectious strains.^70^ This hypothesis is in line with the idea that transmission of environmental mycobacteria depends on the overlapping habitats of these pathogenic strains and humans. Identifying this type of ecological process is now necessary and should allow the infectious pathways of the disease to be clearly determined. Although the ongoing improvement of Next-Generation Sequencing (NGS) technologies may allow for genome-wide screening, SNP methods rely on pure bacterial cultures, and some limitations still remain. First, MU is a slow-growing mycobacterium that is isolated after several months of culturing. Second, VNTR analysis has revealed that pure cultures constrain the isolation to only certain cultivable strains, which exhibit restricted genetic variability compared with the heterogeneity found in the environment.^70^ Finally, only one study has achieved the isolation of MU from an aquatic insect.^71^ We suggest that future investigations should focus on these main limitations.

Environmental MU strains clearly exhibit genetic variability, even among isolates from the same geographic area. Although the majority of studies have focused on clinical isolates obtained from patient biopsies, it is time to obtain a more detailed picture of the genetic diversity of environmental strains, as well as to identify the spatio-temporal distribution and abundances of these infectious strains for humans in addition to the ecological and evolutionary processes underlying the observed patterns. This novel approach to the ecology of BU emergence would improve understanding and monitoring of the infection risk in different regions and should elucidate why only certain specific areas are endemic, even though mycobacteria are widely distributed in the environment.

## CONCLUSIONS

**Figure 4:**
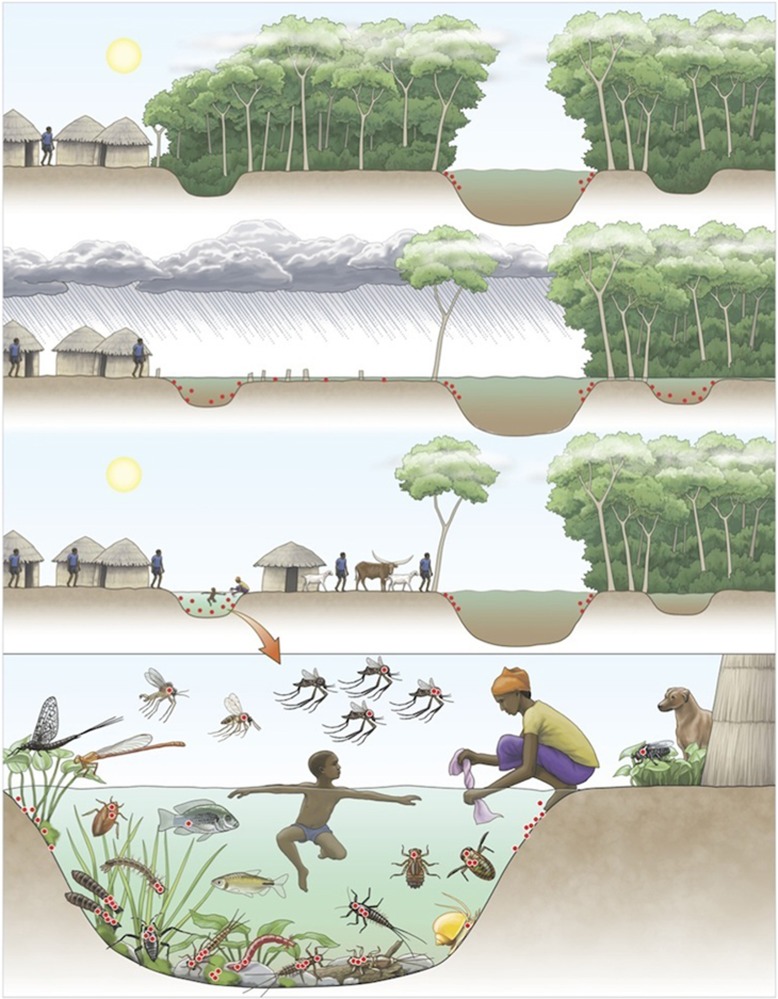
Schematic representation of the links between land-use changes and climatic patterns favoring the emergence of *Mycobacterium ulcerans* (MU) in the environment. Red dots represent the distribution of MU in the environment (panels 1, 2 and 3) as well as among host carrier species communities (panel 4). The top panel shows a pristine ecosystem with MU in low abundance within the aquatic ecosystem. However, in the second panel, deforestation and climatic events (for example, heavy rain) result in intensive flooding and redistribution of MU in the ecosystem. On the left riverbank, the forest has been cut down, whereas on the right, the ecosystem remains pristine.The third panel shows water receding, thus allowing for the formation of small oxbows, which (when the trees have been cut down) are subjected to higher temperatures, higher biofilm development and lower pH and oxygen levels (conditions that are prone to cause the bacterial proliferation). MU is not established in the shaded oxbow. When associated with an increase in contact rates with humans due to a change in land use, the infectious risk of BU becomes greater. The bacterial distribution in the environment and within a high diversity of hosts suggests multiple routes of transmission to humans. The position of aquatic hosts in the water column illustrates their trophic level, from low trophic level organisms (for example, grazing invertebrates) to higher trophic level organisms (for example, fish). Aquatic organisms of a low trophic level generally present greater bacterial loads compared with organisms of a higher trophic level; zero dots represent the absence of MU, and four dots represent a high MU load. Illustration by Emily S. Damstra.

Since the WHO launched the GBUI in 1998, many studies have focused on two research priorities: (i) the ecology of BU and (ii) the mode(s) of transmission of MU to humans. Despite the substantial information provided by these numerous studies, the etiology of BU is currently poorly understood by many authors. The scope of this review was first to show that findings have clearly characterized the multi-host transmission dynamics of MU within the environment through complex global to local interactions. Consequently, its infectious pathways to humans result from a response of the pathogen to universal ecological drivers (for example, biodiversity alteration, land-use changes and climatic patterns enhancing the pathogen's prevalence in the environment) but under the influence of local drivers (for example, abiotic and biotic conditions and behaviors favoring contact with humans). This phenomenon explains why the exact mode of transmission to humans is still unknown: there is more than one mode relying on (i) environmental factors (for example, trophic structure, abiotic parameters and disturbance level), (ii) human host conditions (for example, behavior, immunity and susceptibility) and (iii) MU strains (for example, genetic background and mycolactone type). Here we show that the BU epidemiology must be understood from a global ecological perspective, in which multiple factors act together to maintain the pathogen in the environment and enhance its emergence under specific environmental and human conditions. These favorable environments are now well known to rely on the association between human activities (for example, deforestation and agricultural land-use changes) and climatic patterns (for example, rainfall events) that lead to modified environments and changes in the ecological function of wetland ecosystems. These environmental disturbances are compiled and illustrated in Figure 4. Although the BU emergence events need to be further investigated (Table 2), the interface between (i) changes in the ecological niche of such environmentally persistent microbes, (ii) the overall increased contact between contaminated environments and humans as a result of anthropogenic activities, and (iii) the overlapping habitats of these infectious strains and susceptible individuals might constitute a basis of disease emergence. More generally, EIDs must be considered in terms of these ecological perspectives, in which both anthropogenic and natural disturbances having major effects on ecosystem function, wildlife and human health.

## Figures and Tables

**Table 1:** Taxa reported to be positive for MU DNA and their corresponding functional feeding strategies and food types

**Class**	**Order**	**Feeding strategy**^a^	**Food type**^a^
Plantae (terrestrial)		Photosynthesis	Inorganic nutrients
Plantae (aquatic)	Lamiales	Photosynthesis	Inorganic nutrients
	Alismatales	Photosynthesis	Inorganic nutrients
	Arales	Photosynthesis	Inorganic nutrients
Insecta	Diptera	Gathering collector	Omnivorous
	Hemiptera	Predator/scraper/scavenger	Macroinvertebrate/macroorganism
	Lepidoptera	Predator/scavenger	Phytophagous
	Odonata	Predator	Macroinvertebrate
	Ephemeroptera	Scraper/gathering collector	Detritus/microphyte
	Coleoptera	Predator/scavenger/gathering collector	Detritus/microphyte/microinvertebrate/omnivorous
Arachnida	Acari	Predator	Omnivorous
	Araneae	Predator	Macroinvertebrate
Annelida	Hirudinea	Predator/scavenger	Parasitic
	Oligochaeta	Predator/scavenger	Parasitic
Malacostraca	Decapoda	Predator/scraper	Macroinvertebrate/microphyte
Brachiopoda	Cladocera	Filtering collector	Microorganism
Ostracoda		Gathering collector	Detritus/microphyte
Bivalva	Sphaeriidae	Filtering collector	Phytophagous
	Corbiculidae	Filtering collector	Phytophagous
Gastropoda	Basommatophora	Scraper	Phytophagous/microphyte
	Caenogastropoda	Gathering collector	Detritus/microphyte
Reptilia	Testudines	Predator	Macroorganism
Amphibia	Anura	Predator	Omnivorous
Actinopterygii	Perciformes	Predator/scavenger	Omnivorous/macroinvertebrate
	Cyprinodontiformes	Predator/scavenger	Omnivorous
	Siluriformes	Predator/scavenger	Omnivorous
	Characiformes	Predator/scavenger	Omnivorous
Mammalia	Perissodactyla		Herbivorous
	Carnivora		Carnivorous
	Diprotodontia		Folivore
	Artiodactyla		Herbivorous
	Rodentia		Granivorous

Studies were conducted between 1984 and 2015 from countries where Buruli ulcer cases have been reported. Supporting information is provided in Supplementary Table S1.

a From Morris *et al.*^15^

**Table 2:** Future research areas

**Research areas**	**Scientific objectives**	**Control strategies**
*MU genetics*		
Develop routine laboratory protocols to isolate environmental strains of MU in pure cultures	Allow genome-wide screening of SNPs	Reveal low versus high genetic polymorphisms, with implications for antibiotherapy or vaccine strategies
Identify the various strains of MU co-circulating in the environment, as well as their spatio-temporal dynamics among communities of hosts	Understand the role of the environmental heterogeneity of strains in MU infections	Show the importance of characterizing the infectious risks depending on the spatial heterogeneity, for example, high-risk hot-spots versus low-risk areas

*Human hosts*		
Determine the local immune responses and genetic background in human populations, as well as the resistance/susceptibility to MU	Understand why individuals are susceptible or immune to MU infection. Characterize the immuno-genetic patterns of human populations	Allow for the development of vaccine strategies or inhibitory drug molecules

*Host–MU interactions in animal communities*		
Investigate the relationships between host species and MU	Determine the ecological (for example, symbiotic, parasitic) and molecular processes involved in host–MU interactions in the environment. Understand the patterns and processes involved in MU persistence and distribution in the environment	Characterize areas at higher or lower risk of transmission to humans
Investigate the effects of newly introduced or invasive aquatic species on MU ecology and spread due to socio-economic activities	Determine the effects of invasive or introduced species on the MU ecological niche and ecosystem shifts	Monitor species invasion and introduction as well as their effects on MU spread (for example, aquaculture in Japan)

*Environmental persistence of MU*		
Investigate the role of ecological succession and several disturbance stages in MU prevalence in the environment	Characterize the effects of environmental disturbance on MU dynamics and spread	Monitor environmental disturbances and develop early warning systems to predict BU outbreaks
Investigate the different types and quality of soil found in endemic regions compared with non-endemic areas	Identify the role of physico-chemical soil components and their interactions with MU persistence	Gain a better understanding of the abiotic parameters and produce spatial maps of disease risk areas
Assess the role of pH as an important parameter in MU growth, as well as other chemical and physical parameters	Identify the role of pH and its variability in space and time in water bodies prone to MU growth	Gain a better understanding of the abiotic parameters and produce spatial maps of disease risk areas
Describe the relationships between aquatic plant communities and MU ecology	Study the roles of plants (biofilm) on MU environmental persistence and spread to animal communities. Characterize endemic plant species more favorable for MU growth and the underlying mechanisms	Characterize areas at higher or lower risk of MU growth and transmission to humans
