# Supplementary material for: Global and local environmental changes as drivers of Buruli ulcer emergence
Source: Emerg Microbes Infect. 2017 Apr 26;6(4):e22–. doi: 10.1038/emi.2017.7 (PMC5457673; doi:10.1038/emi.2017.7)
Supplement: Supplementary Table S1 [file emi20177x1.docx]

**Supporting Information**

**Supplementary Table S1.** Taxa reported positives for MU DNA and their corresponding functional feeding strategies and food types. Studies were conducted between 1984-2015 from countries where Buruli ulcer cases have been reported.

| **Class** | **Order** | **Family** | **Species/Genus** | **Stage** | **Feeding strategy^*^** | **Food type^*^** | **References** |
| --- | --- | --- | --- | --- | --- | --- | --- |
| **Plantae (terrestrial)** |  |  |  |  | Photosynthesis | Inorganic nutrients | [1] |
| **Plantae (aquatic)** |  |  |  |  | Photosynthesis | Inorganic nutrients | [1] |
|  | Lamiales | Scrophulariaceae | *Bacopa* |  | Photosynthesis | Inorganic nutrients | [2,3] |
|  | Alismatales | Juncaginaceae | *Triglochin sp.* |  | Photosynthesis | Inorganic nutrients | [4] |
|  | Arales | Araceae | *Montrichardia arborescens* |  | Photosynthesis | Inorganic nutrients | [5] |
| **Insecta** | Diptera |  |  | Larvae | Gathering collectors | Omnivorous | [6,7] |
|  |  | Culicidae |  | Larvae, Adult | Gathering collectors | Omnivorous | [8,9,10] |
|  |  |  | *Aedes aegypti* | Larvae | Gathering collectors | Omnivorous | [11] |
|  |  |  | *Aedes camptorhynchus* | Larvae | Gathering collectors | Omnivorous | [12,13,14] |
|  |  |  | *Aedes notoscriptus* | Larvae | Gathering collectors | Omnivorous | [12] |
|  |  |  | *Coquillettidia linealis* | Larvae | Gathering collectors | Omnivorous | [12] |
|  |  |  | *Culex sp.* | Larvae | Gathering collectors | Omnivorous | [13] |
|  |  |  | *Culex australicus* | Larvae | Gathering collectors | Omnivorous | [12] |
|  |  |  | *Culex restuans* | Larvae | Gathering collectors | Omnivorous | [13] |
|  |  |  | *Anopheles sp.* | Larvae | Gathering collectors | Omnivorous | [12,13] |
|  |  |  | *Toxorhynchites rutilus* | Larvae | Gathering collectors | Omnivorous | [11] |
|  |  | Ceratopogonidae |  | Larvae, Adult | Gathering collectors | Omnivorous | [8,10] |
|  |  | Chironomidae |  | Larvae | Gathering collectors | Omnivorous | [8,9] |
|  |  | Simuliidae |  | Larvae | Gathering collectors | Omnivorous | [8] |
|  |  | Tabanidae |  | Larvae | Gathering collectors | Omnivorous | [8] |
|  |  | Tanypodinae |  | Larvae | Gathering collectors | Omnivorous | [8] |
|  |  | Psychodidae |  | Adult | Gathering collectors | Omnivorous | [9,10] |
|  |  | Sciomycidae |  | Adult | Gathering collectors | Omnivorous | [9] |
|  |  | Syrphidae |  | Adult | Gathering collectors | Omnivorous | [9] |
|  | Hemiptera |  |  | Larvae | Predators | Macroinvertebrate | [6,7] |
|  |  | Naucoridae | *Naucoris cimicoides* |  | Predators | Macroinvertebrate | [2,15,16,17,18] |
|  |  | Naucoridae |  |  | Predators | Macroinvertebrate | [9,19] |
|  |  | Nepidae |  |  | Predators | Macroorganism | [8,9,20] |
|  |  | Gerridae |  |  | Predators | Macroinvertebrate | [20] |
|  |  |  | *Gerris sp.* |  | Predators | Macroinvertebrate | [8,21] |
|  |  | Belostomatidae | *Belostoma sp.* |  | Predators | Macroorganism | [8,9,11,15] |
|  |  |  | *Appasus sp.* |  | Predators | Macroorganism | [19,20,22] |
|  |  |  | *Lethocerus sp.* |  | Predators | Macroorganism | [20] |
|  |  |  | *Diplonychus sp.* |  | Predators | Macroorganism | [22] |
|  |  | Notonectidae |  |  | Predators | Macroorganism | [9,20] |
|  |  | Macrovelidae |  |  | Predators | Macroorganism | [8] |
|  |  | Corixidae |  |  | Scraper | Phytophagous | [8] |
|  |  | Veliidae |  |  | Predator/scavenger | Microinvertebrate | [8] |
|  | Lepidoptera |  |  | Larvae | Predator/scavenger | Phytophagous | [6] |
|  |  | Crambidae |  |  | Predator/scavenger | Phytophagous | [9] |
|  | Odonata |  |  | Larvae | Predators | Macroinvertebrate | [6,7] |
|  |  | Gomphidae |  |  | Predators | Macroinvertebrate | [18] |
|  |  | Libellulidae |  |  | Predators | Macroinvertebrate | [8,9,18] |
|  |  | Protoneuridae |  |  | Predators | Macroinvertebrate | [8,9] |
|  |  | Aeshnidae |  |  | Predators | Macroinvertebrate | [8] |
|  |  | Coenagrionidae |  |  | Predators | Macroinvertebrate | [8] |
|  | Ephemeroptera |  |  | Larvae | Predator/scavenger | Phytophagous | [6] |
|  |  | Baetidae |  |  | Scraper | Microphyte | [8,9] |
|  |  | Caenidae |  |  | Gathering collectors | Detritus/Microphyte | [8,9] |
|  |  | Leptophlebiidae |  |  | Scraper | Detritus/Microphyte | [8] |
|  | Coleoptera |  |  | Larvae, Adult |  |  | [6,7] |
|  |  | Hydrophilidae |  |  | Predator/scavenger | Detritus/Microphyte | [9,19] |
|  |  | Dytiscidae |  |  | Predators | Macroorganism | [8,9] |
|  |  | Elmidae |  |  | Gathering collectors | Detritus/Microphyte | [8,9] |
|  |  | Noteridae |  |  | Predators | Microinvertebrate | [8,9] |
|  |  | Scirtidae |  |  | Predators | Detritus/Microphyte | [9] |
|  |  | Hydraenidae |  |  | Predators | Detritus/Microphyte | [9] |
|  | Plecoptera |  |  | Larvae | Predator/scavenger | Omnivorous | [7] |
| **Arachnida** | Acari | Hydracarina |  |  | Predators | Omnivorous | [6] |
|  | Araneae |  |  |  | Predators | Macroinvertebrate | [6,7,8,9] |
| **Annelida** | Hirudinea |  |  |  | Predator/scavenger | Parasitic | [6,8] |
|  | Oligochaeta |  |  |  | Predator/scavenger | Parasitic | [6,8,9] |
| **Malacostraca** | Decapoda |  |  |  | Predators | Macroinvertebrate | [6,7] |
|  |  | Cambaridae | *Cambaroides japonicus* |  | Predators | Macroinvertebrate | [23] |
|  |  | Euryrhynchidae | *Euryhynchus amazoniensis* |  | Predators | Macroinvertebrate | [8] |
|  |  | Palaemonidae |  |  | Scraper | Microphyte | [8] |
| **Branchiopoda** | Cladocera |  |  |  | Filtering collectors | Microorganism | [6] |
| **Ostracoda** |  |  |  |  | Gathering collectors | Detritus/Microphyte | [8,9] |
| **Bivalva** |  |  |  |  | Filtering collectors | Phytophagous | [6] |
|  | Sphaeriidae |  |  |  | Filtering collectors | Phytophagous | [9] |
|  | Corbiculidae |  |  |  | Filtering collectors | Phytophagous | [9] |
| **Gastropoda** |  |  |  |  | Scraper | Phytophagous | [6] |
|  | Basommatophora | Planorbidae | *Bulinus senegalensis* |  | Scraper | Phytophagous | [19] |
|  |  |  | *Bulinus sp.* |  | Scraper | Phytophagous | [9,18] |
|  |  |  | *Planorbis planorbis* |  | Scraper | Phytophagous | [18] |
|  |  | Ancylidae |  |  | Scraper | Microphyte | [8] |
|  |  | Physidae |  |  | Scraper | Phytophagous | [8,9] |
|  | Caenogastropoda | Ampullariidae | *Pomacea canaliculata* |  | Gathering collectors | Detritus/Microphyte | [18] |
| **Reptilia** | Testudines | Trionychidae | *Lissemys punctata* |  | Predators | Macroorganism | [24] |
| **Amphibia** | Anura |  |  | Tadpole, Adult | Predators | Omnivorous | [6,7,8,9] |
| **Actinopterygii** |  |  |  |  | Predator/scavenger | Omnivorous | [6,7] |
|  | Perciformes | Cichlidae | *Sarotherodon galilaeus* |  | Predator/scavenger | Omnivorous | [25] |
|  |  |  | *Hemichromis bimaculatus* |  | Predator/scavenger | Omnivorous | [25] |
|  |  |  | *Tilapia sp.* |  | Predator/scavenger | Omnivorous | [19] |
|  |  |  | *Krobia guianensis* |  | Predator/scavenger | Omnivorous | [8] |
|  |  | Polycentridae | *Polycentrus punctatus* |  | Predator/scavenger | Macroinvertebrate | [8] |
|  | Cyprinodontiformes | Aplocheilidae | *Epiplatys bifasciatus* |  | Predator/scavenger | Omnivorous | [25] |
|  |  | Poeciliidae | *Aplocheilichthys sp.* |  | Predator/scavenger | Omnivorous | [25] |
|  |  | Rivulidae | *Rivulus lungi* |  | Predator/scavenger | Omnivorous | [8] |
|  | Siluriformes | Clariidae |  |  | Predator/scavenger | Omnivorous | [18] |
|  | Characiformes | Lebiasinidae | *Copella carsevennensis* |  | Predator/scavenger | Omnivorous | [8] |
|  |  |  | *Pyrrhulina filamentosa* |  | Predator/scavenger | Omnivorous | [8] |
|  |  | Characidae | *Hemigrammus rodwayi* |  | Predator/scavenger | Omnivorous | [8] |
|  |  |  | *Hemigrammus unilineatus* |  | Predator/scavenger | Omnivorous | [8] |
|  |  |  | *Pristella maxilaris* |  | Predator/scavenger | Omnivorous | [8] |
| **Mammalia** | Perissodactyla | Equidae | *Equus caballus* |  |  | Herbivorous | [26] |
|  | Carnivora | Felidae |  |  |  | Carnivorous | [27] |
|  | Diprotodontia | Pseudocheiridae | *Pseudocheirus peregrinus* |  |  | Folivore | [1] |
|  |  | Phalangeridae | *Trichosurus vulpecula* |  |  | Folivore | [1] |
|  |  | Phascolarctidae | *Phascolarctos cinereus* |  |  | Folivore | [28] |
|  | Artiodactyla | Camelidae | *Vicugna pacos* |  |  | Herbivorous | [29] |
|  | Rodentia | Muridae | *Mastomys* |  |  | Granivorous | [30] |

**^*^** From Morris *et al*. (2016)^31^

[1] Fyfe JAM, Lavender CJ, Handasyde KA et al. A major role for mammals in the ecology of Mycobacterium ulcerans. PLoS Negl Trop Dis 2010; 4: e791.

[2] Marsollier L, Honore N, Legras P et al. Isolation of three Mycobacterium ulcerans strains resistant to rifampin after experimental chemotherapy of mice. Antimicrob Agents Chemother 2003; 47: 1228–1232.

[3] Marsollier L, Stinear T, Aubry J et al. Aquatic plants stimulate the growth of and biofilm formation by Mycobacterium ulcerans in

axenic culture and harbor these bacteria in the environment. Appl Environ Microbiol 2004b, 70, 1097–1103.

[4] Stinear TP, Davies JK, Jenkin GA et al. Identification of Mycobacterium ulcerans in the environment from regions in southeast Australia in

which it is endemic with sequence capture-PCR. Appl Environ Microbiol 2000; 66: 3206–3213.

[5] Morris A, Gozlan RE, Marion E et al. First detection of Mycobacterium ulcerans DNA in environmental samples from South America. PLoS

Negl Trop Dis 2014; 8: 8–13.

[6] Garchitorena A, Roche B, Kamgang R et al. Mycobacterium ulcerans ecological dynamics and its association with freshwater ecosystems and aquatic communities: results from a 12-month environmental survey in Cameroon. PLoS Negl Trop Dis 2014; 8: e2879.

[7] Zogo B, Djenontin A, Carolan K et al. A field study in Benin to investigate the role of mosquitoes and other flying insects in the ecology of Mycobacterium ulcerans. PLoS Negl Trop Dis 2015; 9: 1–12.

[8] Morris AL. Identifying biological and environmental indicators of emerging infectious diseases: the case of Buruli ulcer. Doctoral thesis, Bournemouth University 2015; 204pp.

[9] Williamson HR, Benbow ME, Nguyen KD et al. Distribution of Mycobacterium ulcerans in Buruli ulcer endemic and non-endemic aquatic sites in Ghana. PLoS Negl Trop Dis 2008; 2: e205.

[10] Le Gall P, Landier J, De Matha Ndengué J et al. Détection de Mycobacterium ulcerans chez les arthropodes domestiques dans un site d’endémie de l’uclère de Buruli, Akonolinga au Cameroun. Réunion de l’OMS sur l’uclère de Buruli: lutte et recherche 2015; 1 p.

[11] Wallace JR, Gordon MC, Hartsell L et al. Interaction of Mycobacterium ulcerans with mosquito species: implications for transmission and trophic relationships. Appl Environ Microbiol 2010; 76: 6215–6222.

[12] Johnson PDR, Azuolas J, Lavender CJ et al. Mycobacterium ulcerans in mosquitoes captured during outbreak of Buruli ulcer, southeastern Australia. EmergInfect Dis 2007; 13: 1653–60.

[13] Lavender CJ, Fyfe JAM, Azuolas J et al. Risk of Buruli ulcer and detection of Mycobacterium ulcerans in mosquitoes in Southeastern

Australia. PLoS Negl Trop Dis 2011; 5: 1–6.

[14] Fyfe JAM, Lavender CJ, Johnson PDR et al. Development and application of two multiplex real-time PCR assays for the detection of Mycobacterium ulcerans in clinical and environmental samples. Appl Environ Microbiol 2007; 73: 4733–4740.

[15] Portaels F, Elsen P, Guimaraes-Peres A et al. Insects in the transmission of Mycobacterium ulcerans infection. Lancet 1999; 93: 986.

[16] Marsollier L, Robert R, Aubry J et al. Aquatic insects as a vector for Mycobacterium ulcerans. Appl Environ Microbiol 2002; 68: 4623–8.

[17] Marsollier L, André J-PS, Frigui W et al. Early trafficking events of Mycobacterium ulcerans within Naucoris cimicoides. Cell Microbiol

2007; 9: 347–55.

[18] Marsollier L, Sévérin T, Aubry J et al. Aquatic snails, passive hosts of Mycobacterium ulcerans. Appl Environ Microbiol 2004; 70: 6296

6298.

[19] Kotlowski R, Martin A, Ablordey A et al. One-tube cell lysis and DNA extraction procedure for PCR-based detection of Mycobacterium ulcerans in aquatic insects, molluscs and fish. J Med Microbiol 2004; 53: 927–933.

[20] Marion E, Eyangoh S, Yeramian E et al. Seasonal and regional dynamics of M . ulcerans transmission in environmental context: deciphering the role of water bugs as hosts and vectors. PLoS Negl Trop Dis 2010; 4: e731.

[21] Portaels F, Meyers WM, Ablordey A et al. First cultivation and characterization of Mycobacterium ulcerans from the environment. PLoS Negl Trop Dis 2008; 2: e178.

[22] Mosi L, Williamson H, Wallace JR et al. Persistent association of Mycobacterium ulcerans with West African predaceous insects of the family Belostomatidae. Appl Environ Microbiol 2008; 74: 7036–7042.

[23] Luo Y, Degang Y, Ohtsuka M et al. Detection of Mycobacterium ulcerans subsp. Shinshuense DNA from a water channel in familial Buruli ulcer cases in Japan. Future Microbiol 2015; 10: 461–469.

[24] Sakaguchi K, Iima H, Hirayama K et al. Mycobacterium ulcerans infection in an Indian flap-shelled turtle (Lissemys punctata punctata). J VetMed Sci 2011; 73: 1217–1220.

[25] Eddyani M, Ofori-adjei D, Teugels G et al. Potential role for fish in transmission of Mycobacterium ulcerans disease (Buruli Ulcer): an

environmental study. Appl Environ Microbiol 2004; 70: 5679–5681.

[26] van Zyl A, Daniel J, Wayne J et al. Mycobacterium ulcerans infections in two horses in south-eastern Australia. Australian Vet J 2010; 88: 101–106.

[27] Elsner L, Wayne J, O’Brien CR et al. Localised Mycobacterium ulcerans infection in a cat in Australia. J Feline Med Surg 2008; 10: 407–412.

[28] Mitchell PJ, Jerrett IV, Slee KJ. Skin ulcers caused by Mycobacterium ulcerans in koalas near Bairnsdale, Australia. Pathology 1984; 16: 256–260.

[29] O’Brien C, Kuseff G, McMillan E et al. Mycobacterium ulcerans infection in two alpacas. Australian Vet J 2013; 91: 296–300.

[30] Narh CA, Mosi L, Quaye C et al. Source tracking Mycobacterium ulcerans infections in the Ashanti region, Ghana. PLoS Negl Trop Dis 2015; 9: e0003437.

[31] Morris A, Guégan J-F, Benbow ME et al. Functional diversity as a new framework for understanding the ecology of an emerging generalist pathogen. EcoHealth 2016; DOI: 10.1007/s10393-016-1140-x.
