# Supplementary material for: Global and local environmental changes as drivers of Buruli ulcer emergence
Source: Emerg Microbes Infect. 2017 Apr 26;6(4):e22–. doi: 10.1038/emi.2017.7 (PMC5457673; doi:10.1038/emi.2017.7)
Supplement: Supplementary Table S2 [file emi20177x2.docx]

**Supporting Information**

**Supplementary Table S2.** Trophic levels determined from stable isotope analyses and average bacterial load (i.e. bacteria per mg of organism) estimated for each taxa of the aquatic community found positives for IS*2404* and KR from 17 sites sampled in French Guiana (South America). Data were provided by Morris et al. (2016)^22^ and the average bacterial load was calculated from organisms belonging to the same taxa. Data were transformed using the square root mean number of bacteria (SQRT). The taxa presented here are arranged in ascending trophic levels.

| **Taxa** | **δ^13^C** | **δ^15^N** | **Mean bacterial load** | **SQRT** |
| --- | --- | --- | --- | --- |
| Gerridae | -38.11 | 6.16 | 17.68 | 4.2 |
| Veliidae | -38.11 | 6.16 | 3543.21 | 59.52 |
| Simuliidae | -36.33 | 4.07 | 5224.68 | 72.28 |
| *Polycentrus punctatus* | -36.33 | 7.93 | 13.3 | 3.65 |
| Chironomidae | -35.27 | 4.73 | 1604.4 | 40.05 |
| Baetidae | -35.05 | 5.15 | 4050.15 | 63.64 |
| Oligochaeta | -34.94 | 3.07 | 2618.9 | 51.18 |
| Polymitarcyidae | -34.94 | 3.07 | 10385.77 | 101.91 |
| Caenidae | -34.75 | 5.15 | 3574.8 | 59.79 |
| Leptophlebiidae | -34.75 | 5.15 | 2641.27 | 51.39 |
| Corixidae | -34.21 | 5.37 | 3840.51 | 61.97 |
| Ceratopogonidae | -32.57 | 3.56 | 1393.64 | 37.33 |
| Palaemonetes | -31.92 | 7.04 | 227.5 | 15.08 |
| Coenagrionidae | -31.8 | 11.65 | 1044.78 | 32.32 |
| Aeshnidae | -31.55 | 5.12 | 18.43 | 4.29 |
| Libellulidae | -31.55 | 5.12 | 866.76 | 29.44 |
| Tanypodinae | -31.04 | 6.94 | 1251.15 | 35.37 |
| *Hemigrammus rodwayi* | -30.88 | 7.41 | 22.25 | 4.72 |
| Dyticidae (larvae) | -30.62 | 4.56 | 20.41 | 4.52 |
| *Pristella maxilaris* | -30.36 | 8.26 | 18.06 | 4.25 |
| Noteridae (larvae) | -30.28 | 4.97 | 150.02 | 12.25 |
| *Krobia guianensis* | -30.04 | 10.53 | 68.8 | 8.29 |
| Anura | -29.91 | 5.53 | 191.39 | 13.83 |
| *Euryhynchus amazoniensis* | -29.89 | 8.01 | 135.55 | 11.64 |
| Belostomatidae | -29.57 | 5.48 | 24.47 | 4.95 |
| Tabanidae | -29.26 | 5.49 | 226.09 | 15.04 |
| *Rivulus lungi* | -28.91 | 8.21 | 174.09 | 13.19 |
| Macrovelidae | -28.88 | 3.12 | 1424.31 | 37.74 |
| Ostracoda | -28.73 | 2.19 | 718.92 | 26.81 |
| *Pyrrhulina filamentosa* | -28.66 | 7.67 | 1.54 | 1.24 |
| Protoneuridae | -28.62 | 7.53 | 153.49 | 12.39 |
| *Hemigrammus unilineatus cayennensis* | -28.5 | 7.75 | 6.88 | 2.62 |
| Dytiscidae (adult) | -28.03 | 4.22 | 249.79 | 15.8 |
| *Copella carsevennensis* | -27.12 | 7.85 | 302.76 | 17.4 |
| Noteridae (adult) | -27.09 | 3.09 | 2297.49 | 47.93 |
| Araneae | -26.92 | 7.65 | 62.47 | 7.90 |
| Nepidae | -26.56 | 11.13 | 56.64 | 7.53 |
| Elmidae | -26.19 | 3.74 | 1188.26 | 34.47 |
| Hirudinea | -25.45 | 9.65 | 17.86 | 4.23 |
| Planorbidae | -18.64 | 2.32 | 1085.58 | 32.95 |
| Physidae | -12.96 | 0.99 | 261.11 | 16.16 |
